# Supplementary material for: Characterization of Vaginal Microbiota in Third Trimester Premature Rupture of Membranes Patients through 16S rDNA Sequencing
Source: Pathogens. 2022 Jul 28;11(8):847. doi: 10.3390/pathogens11080847 (PMC9413980; doi:10.3390/pathogens11080847)
Supplement: Supplementary file 1 [file pathogens-11-00847-s001.zip › pathogens-1839034-supplementary.pdf]

Supplementary Materials

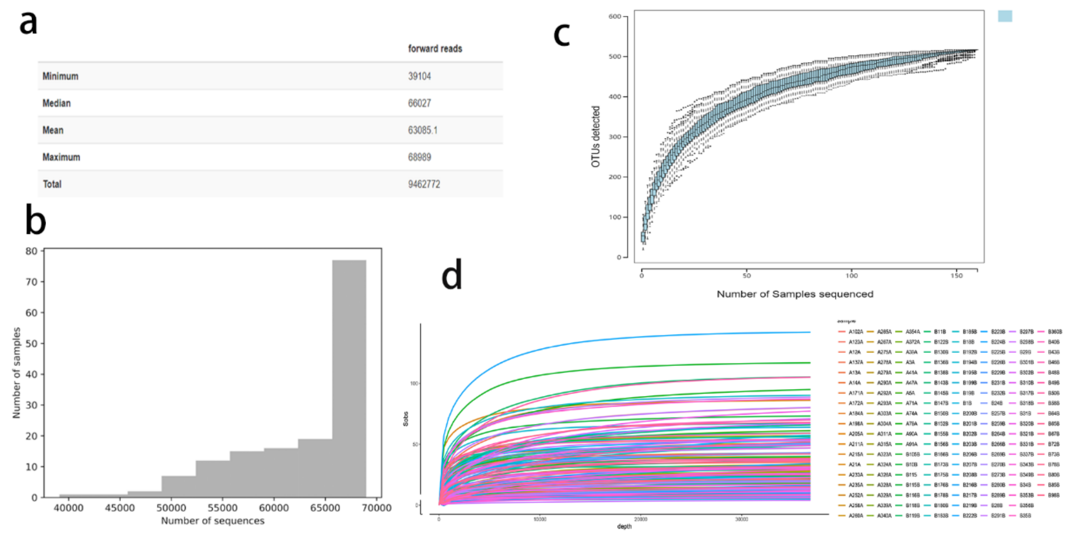

Figure S1. 16S rDNA V3-V4 gene sequencing quantity control.
(a) Reads statistics of all samples. (b) Reads distribution of all samples. Most of the samples had reads between 50,000-68,000. (c) The Species dilution curve indicated that the number of samples was enough for the study. (d) The Sobs Diversity Rarefaction curve showed the sequencing depth per sample.

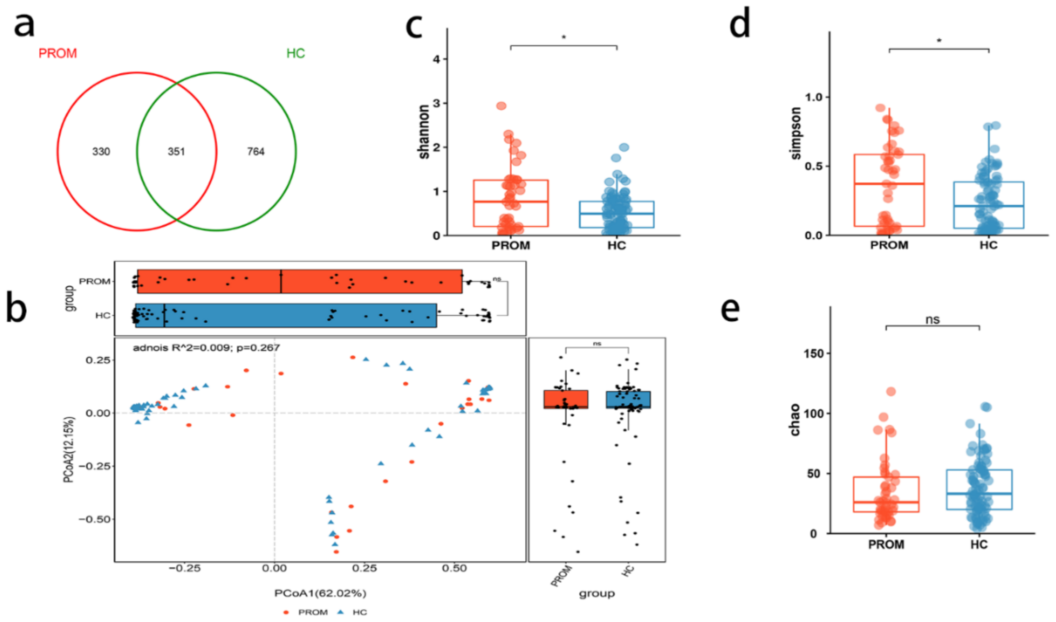

Figure S2. Characteristics of flora composition between PROM and HC groups. (a) Distribution of OTUs showed in Venn graph. (b) PCoA analysis showed no difference between the two groups. (c) The Shannon index in the PROM group was higher than the HC group (*P*=0.014). (d) The Simpson index in the PROM group was higher than the HC group (*P*=0.014). (e) The Chao index showed no statistical difference between the two groups.

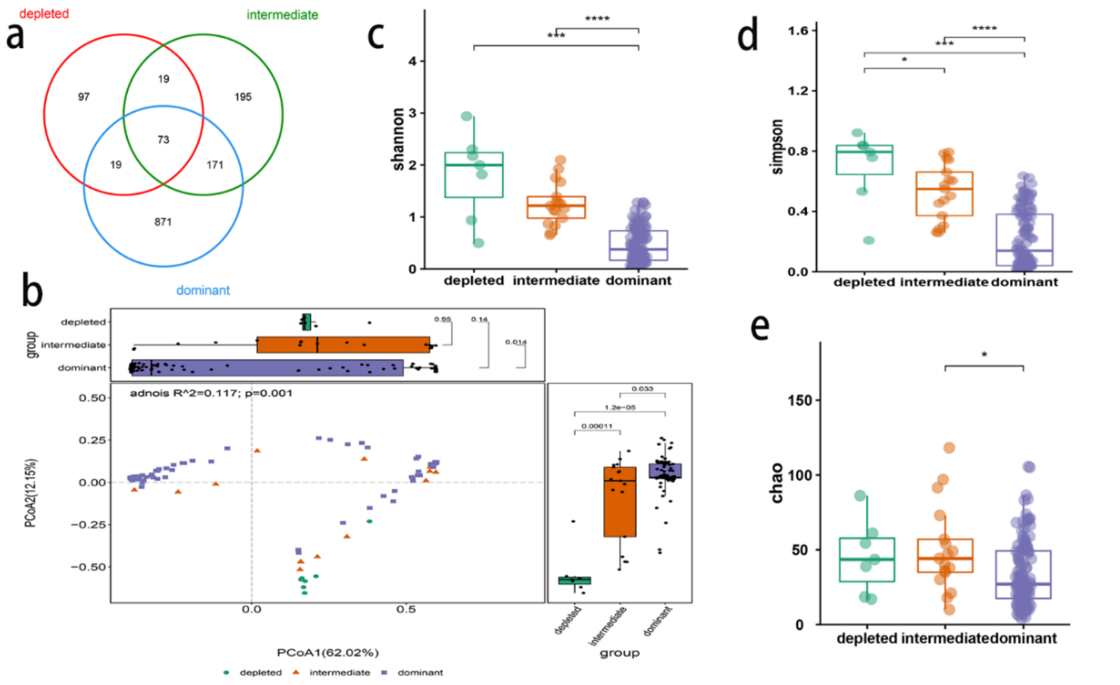

Figure S3. Characteristics and diversity of vaginal microbiome composition among the dominant group, the intermediate group, and the depleted group.

(a) Distribution of OTUs among the three groups showed in Venn graph. (b) The Principal Co-ordinates Analysis (PCoA) showed statistical differences among the three groups. (c) The Shannon index showed statistical differences between the depleted group and the dominant group (*P*<0.001), as well as between the intermediate group and the dominant group (*P*<0.0001). (d) The Simpson indexes showed statistical differences between any two groups (the depleted group vs. the intermediate group P=0.047, the depleted group vs. the dominant group *P*<0.001, the intermediate group vs. the dominant group *P*<0.0001, respectively). (e) The Chao index showed a statistical difference between the intermediate group and the dominant group (*P*=0.02).

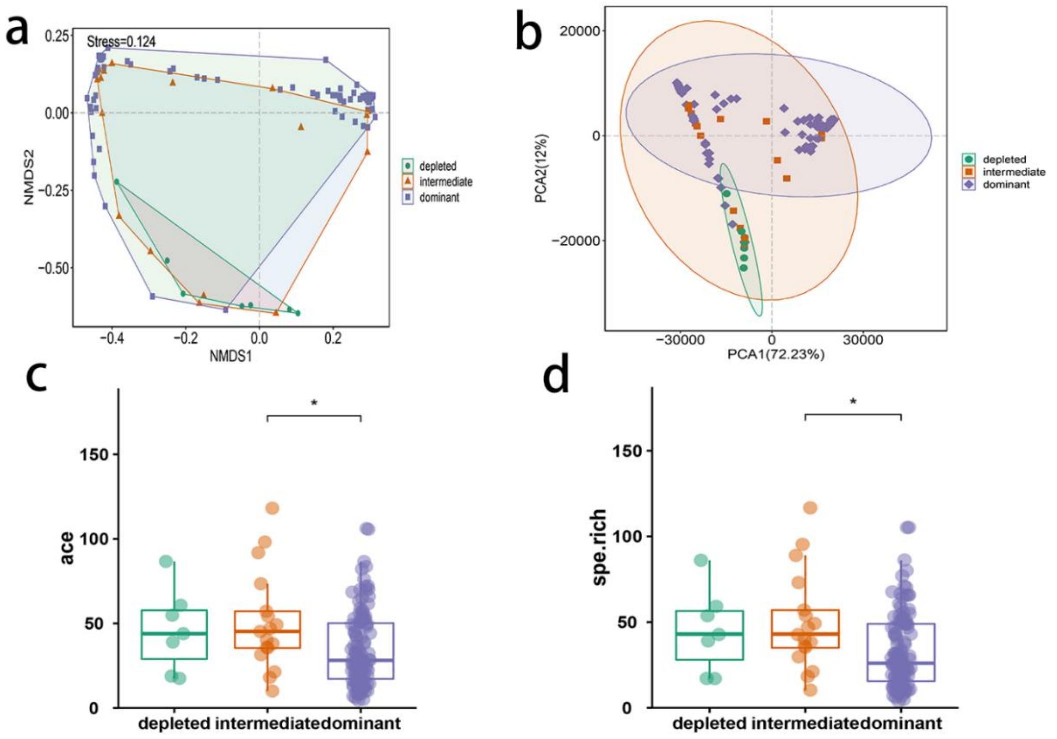

Figure S4. Dimensionality reduction analysis and alpha diversity indexes in the dominant group, the intermediated group, and the depleted group.

The dimensionality reduction analysis was performed to compare the principal component among the three groups, NMDS1 (a) and PCA (b). The Ace index (c) and Species Richness index (d) showed statistical differences between the intermediate and the dominant groups (*P*=0.07, *P*=0.04 respectively).

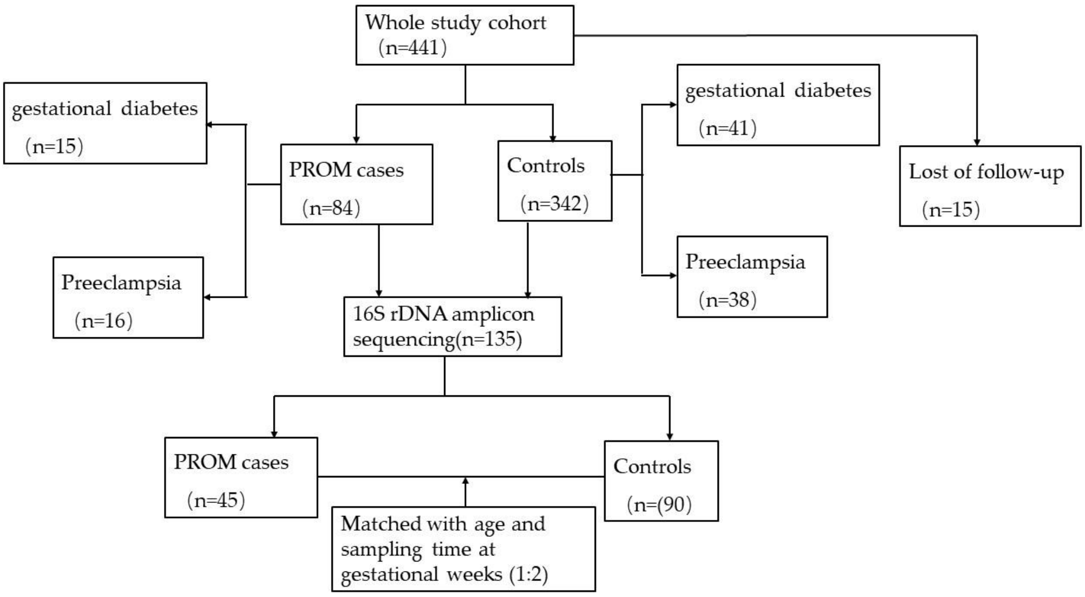

Figure S5. Patient selection flowchart for this study.
